# Assessing the prognostic impact of prostatic urethra involvement and developing a nomogram for T1 stage bladder cancer

**DOI:** 10.1186/s12894-023-01342-2

**Published:** 2023-11-10

**Authors:** Hao Wan, Xiangpeng Zhan, Xuwen Li, Tao Chen, Xinxi Deng, Yang Liu, Jun Deng, Bin Fu, Yu Li

**Affiliations:** 1 Department of Urology, The First Affiliated Hospital of Nanchang University, Nanchang, Jiangxi Province China; 2 Department of Cardiology, The Second Affiliated Hospital of Nanchang University, Nanchang, Jiangxi Province China; 3 Department of Urology, Jiu Jiang NO.1 People’s Hospital, Jiujiang, Jiangxi Province China

**Keywords:** Prognosis, T1 bladder cancer, Prostatic urethra involved, SEER, Nomogram

## Abstract

**Purpose:**

To investigate prognostic values of prostatic urethra involvement (PUI) and construct a prognostic model that estimates the probability of cancer-specific survival for T1 bladder cancer patients.

**Method and materials:**

We investigated the national Surveillance, Epidemiology, and End Results (SEER) database (2004–2015) to get patients diagnosed with T1 bladder cancer. An external validation cohort was obtained from the First Affiliated Hospital of Nanchang University. The Kaplan–Meier method with the log-rank test was applied to assess cancer-specific survival (CSS) and overall survival (OS). Moreover, the propensity score matching (PSM) and multivariable Cox proportional hazard model were performed. All patients were randomly divided into the development cohort and validation group at the ratio of 7:3. The performance of the model was internally validated by calibration curves and the concordance index (C-index).

**Results:**

The PUI group had a lower survival rate of both CSS and overall survival OS before and after PSM when compared to non-involved patients (All *P* < 0.05). Multivariate analysis revealed a poor prognosis in the PUI group for cancer-specific mortality (CSM) and all-cause mortality (ACM) analyses before and after PSM (All *P* < 0.05). Seven variables, including age, surgery, radiotherapy, tumour size, PUI, and marital status, were incorporated in the final nomogram. The C-index in the development cohort was 0.715 (0.711–0.719), while it was 0.672 (0.667–0.677) in the validation group. Calibration plots for 3- and 5-year cancer-specific survival showed good concordance in the development and validation cohorts.

**Conclusions:**

PUI was an independent risk factor of ACM and CSM in T1 bladder cancer patients. In addition, a highly discriminative and precise nomogram that predicted the individualized probability of cancer-specific survival for patients with T1 bladder cancer was constructed.

**Supplementary Information:**

The online version contains supplementary material available at 10.1186/s12894-023-01342-2.

## Introduction

Urothelial bladder cancer (UBC) was the 7th most common cancer in men and the 17th most common in women worldwide, and it was also the fourth and ninth most common malignancy in men and women, respectively, in the Western world [1]. In all patients diagnosed with bladder cancer, about 75% of patients were confirmed as non-muscle-invasive bladder cancer (NMIBC), and 25% of NMIBC patients presented as T1 stage with an invasion of the subepithelial connective tissue, which is the lamina propria [2, 3]. Compared with other styles of NMIBC, T1 bladder cancer (BC) was common high grade and more likely to progress locally and spread outside the bladder, which have a 77% 10-year recurrence rate, a 42% 10-year progression rate, and 15%10-year cancer-specific mortality (CSM) according to a review [4].

For the majority of patients diagnosed with High-grade T1 (HGT1) bladder cancer, the initial and preferred treatment option is bladder preservation therapy using bacillus Calmette-Gue’rin (BCG) [3, 5]. Nevertheless, there is ongoing debate regarding the choice between conservative therapy and early radical cystectomy due to the high rate of disease progression in HGT1 tumors and unsatisfactory long-term effectiveness of BCG [6]. In order to make more informed decisions, several prognostic factors have been investigated to assess the prognosis of patients with HGT1 bladder cancer, including tumor grade, stage, previous recurrences, tumor size, multifocality, and the presence of carcinoma in situ (CIS) [7]. Similarly, some studies suggested that the prostatic urethra might be a sanctuary site where tumours have less exposure to intravesical therapy [8, 9]. The efficacy of intravesical therapy was undermined, and it might cause a higher recurrence rate. In addition, the prostatic urethra was a part of the urethra, which was close to the bladder. Following the direction of urine flow, the tumour of the bladder might be observed in the urethra, although the condition was rare [10]. Meanwhile, the tumor in the prostatic urethra was hard to accurately stage on account that it might be the early manifestation of metastasis of the prostate. It was difficult to detect unless the entire prostate was removed [11]. However, the studies were focusing on the effect on the prognosis of T1 bladder cancer patients with prostatic urethra involvement (PUI) were still lacking. Therefore, to fully evaluate the prognosis of T1 bladder cancer patients, it is crucial to investigate the impact of PUI on disease progression and recurrence. This gap in knowledge is significant as the prostatic urethra may serve as a sanctuary site for tumours, leading to a higher recurrence rate and reduced efficacy of intravesical therapies. Moreover, accurately staging tumours in the prostatic urethra can be challenging, making early detection and treatment difficult. Understanding the impact of PUI on T1 bladder cancer patients’ prognosis can help inform treatment decisions and improve long-term outcomes. It is thus imperative to explore this aspect in future studies.

A nomogram is a reliable and easily understandable statistical tool that is extensively utilized to generate personalized prognostic information based on key factors identified through screening. By using a prognostic nomogram, we can assess an individual’s prognosis and make informed treatment decisions. This helps in tailoring treatments according to each person’s unique circumstances. Prognostic models, such as the EORTC and CUETO [7, 12], have been developed to assess non-invasive and BCG-treated patients’ prognosis. There were already some prediction models for the prognosis and recurrence of T1 stage bladder cancer. However, the predictive accuracy of these models is relatively insufficient [13–15]. For instance, Fucai Tang et al. used the SEER database to construct a nomogram for predicting CSS in T1 bladder cancer, and the C-indices obtained were 0.700 [14]. Such predictive accuracy is considered relatively inadequate. This might be attributed to the lack of inclusion of more clinically meaningful variables in the model and the absence of external data validation, which compromises the rigor of the results.

We investigated the national Surveillance, Epidemiology, and End Results (SEER) database (2004–2015) to get all patients diagnosed with T1 stage based on American Joint Committee on Cancer (AJCC) 7th edition to understand better the prognostic values of PUI and survival outcomes in T1 bladder cancer patients. Moreover, a prognostic nomogram predicting individualized CSS of T1 bladder cancer patients was established.

## Materials and methods

### Study population

All patients included in this study were sourced from the Surveillance, Epidemiology, and End Results (SEER) database, covering the period from 2004 to 2015. The sample population in this database accurately represents the demographic characteristics and cancer incidence rates of the United States. A total of 19,774 patients were enrolled based on the following inclusion criteria: (1) registration between 2004 and 2015, (2) male gender recorded, (3) diagnosed at the T1 stage according to the 7th edition of the American Joint Committee on Cancer (AJCC) using CS Extension codes C155 and C160, (4) positive confirmation of diagnosis through histology, and (5) histology recorded as transitional cell carcinoma. The following exclusion criteria were also applied: (1) unknown follow-up status, (2) cases labeled as T1 with no additional extension information, (3) stages N1, N2, and N3, and (4) stage M1.

We obtained an external validation cohort to assess the prognostic survival of bladder cancer patients with PUI. This cohort comprised patients diagnosed with bladder cancer at the First Affiliated Hospital of Nanchang University from 2015 to 2021. All these patients were postoperatively diagnosed as stage T1 based on pathological reports. Demographic and clinical data, including age, sex, grade, chemotherapy, survival time, and survival status, were collected.

### Definition of variables and endpoints

Patients with stage T1 bladder cancer were categorized into two groups: the non-involved group and the PUI group. The T1 stage in the non-involved group was defined as the presence of subepithelial connective tissue, including tunica propria, lamina propria, and submucosa, which are part of the bladder stroma. The analysis considered various demographic characteristics such as age at diagnosis, race, and marital status. Tumor characteristics included histology, grade, and tumor size, while treatment characteristics comprised information on surgery, radiotherapy, and chemotherapy. The data pertaining to radiotherapy and chemotherapy were consistent with the data use agreement. Specifically, continuous variables like age were converted into categorical variables, with age groups defined as < 50, 50–60, 60–70, 70–80, and > 80. Tumor size was categorized into four groups: <  = 3 cm, 3-6 cm, > 6 cm, and unknown. Marital status was divided into four categories: married, single, widowed or divorced, and unknown status. The surgical method was determined based on the “RX Summ-Surg Prim Site (1998 +)” column and included transurethral bladder tumor resection (TURBT), partial cystectomy (PC), radical cystectomy (RC), pelvic exenteration (PE), or no surgery. Other variables considered in the study were race (white, black, Asian/Pacific Islander, and American Indian/Alaskan Native), tumor grade (Low, High, Unknown), radiotherapy (no/unknown, yes), and chemotherapy (no/unknown, yes).

The primary endpoints of this study were all-cause mortality (ACM) and cancer-specific mortality (CSM), which were determined based on data from the SEER (Surveillance, Epidemiology, and End Results) database. The Cause of Death Recode in the SEER database was utilized to identify the causes of death, considering both cancer-specific and non-cancer-related factors, as indicated by the ICD 8–10 codes. Cancer-specific mortality (CSM) was recorded specifically for bladder cancer based on the SEER mortality code. All-cause mortality (ACM) encompassed deaths caused by bladder cancer as well as deaths resulting from other causes. Survival time was defined as the duration from initial diagnosis to death from any cause or the last follow-up.

### Statistical analysis

Propensity score matching (PSM) and all statistical analyses were conducted using SPSS version 22.0 (IBM Corp, Armonk, NY) and R statistical software packages (http://www.R-project.org). PSM was performed separately for the non-involved group and the PUI group, with a caliper set at 0.001, using nearest neighbor matching. Two-tailed *p*-values were calculated, and a significance level of *P* < .050 was used. Continuous variables were analyzed using Two-sample t-tests, while categorical variables were analyzed using Pearson’s chi-square tests. Frequency and proportion were used to represent categorical variables. The CSS and OS curves were generated using the adjusted Kaplan–Meier method, with the log-rank test applied.

To create the predictive nomogram, all patients diagnosed with T1 bladder cancer were randomly divided into a development cohort (70%) and a validation cohort (30%) using the “caret” package in R. The development cohort was used to establish the predictive nomogram, and internal validation was performed in the validation cohort. Multivariable Cox regression analysis was used to estimate hazard ratios (HR) and their 95% confidence intervals (95% CI). Subsequently, variables associated with CSS were used to construct the predicted nomogram using the “rms” package. The performance of the prediction model was evaluated using the concordance index (C-index) and calibration curves, with 100 bootstrap resamples.

## Results

### Descriptive characteristics of the study population before and after PSM

In this study, a total of 19,774 T1BC patients were enrolled between 2004 and 2015. Prior to propensity score matching (PSM), 19,520 patients were categorized into the non-involved group, while 254 patients were in the PUI group. Following PSM, 244 patients from the non-involved group were selected. The baseline characteristics of the cohort, both before and after PSM, are summarized in Table 1. No significant differences were observed in terms of age, race, marital status, number of tumors, radiotherapy, and chemotherapy recode between the two groups (*P* > .050). However, the non-involved group had a higher percentage of high-grade tumors compared to the PUI group (86.1% vs 75.6%, *P* < 0.001). Additionally, patients in the PUI group had a slightly higher rate of tumor size > 6cm compared to the non-involved group (3.9% vs 3.5%). The distribution of surgical approaches differed significantly between the two groups (*P* < 0.001), with a greater tendency for radical cystectomy (RC) (4.3% vs 2.2%) and pelvic exenteration (5.9% vs 2.3%) in the PUI group. After 1:1 PSM, adjusting for age, race, marital status, grade, tumor size, surgery, radiotherapy, and chemotherapy, differences in age, grade, and number of tumors still remained between the two groups (*P* < 0.05).

**Table 1:** Clinicopathological features between Non-involved and PUI before and after propensity score matching

	**No PSM**			**PSM**		
**Variables**	**Non-involved (*n* = 19520)**	**PUI (*n* = 254)**	***P-*value**	**Non-involved (*n* = 244)**	**PUI (*n* = 254)**	***P*-value**
**Age (year)**						
Mean ± SD	72.31 ± 10.5	71.9 ± 11.16	0.532	74.26 ± 10.07	71.9 ± 11.16	0.014*
Median (25th–75th percentile)	72(67.5–82)	72(67.5–82)	0.764	77.5(67.5–82)	72(67.5–82)	0.024*
**Race**			0.784			0.215
White	17464(89.5%)	226(89.0%)		205(84.0%)	226(89.0%)	
Black	1128(5.8%)	17(6.7%)		18(7.4%)	17(6.7%)	
AIAN	53(0.3%)	0(0.0%)		1(0.4%)	0(0.0%)	
API	875(4.5%)	11(4.3%)		20(8.2%)	11(4.3%)	
**Marital status**			0.418			0.083
Married	11543(59.1%)	148(58.3%)		144(59.0%)	148(58.3%)	
Single	2059(10.5%)	33(13.0%)		35(14.3%)	33(13.0%)	
Widowed/Divorced	4448(22.8%)	59(23.2%)		40(16.4%)	59(23.2%)	
Unknown	1470(7.5%)	14(5.5%)		25(10.2%)	14(5.5%)	
**Grade**			< 0.001*			< 0.001*
Low	2705(13.9%)	36(14.2%)		13(5.3%)	36(14.2%)	
High	16815(86.1%)	192(75.6%)		231(94.7%)	192(75.6%)	
Unknown	0(0%)	26(10.2%)		0(0.0%)	26(10.2%)	
**Surgery**			< 0.001*			0.154
TURBT	18013(92.3%)	215(84.6%)		211(86.5%)	215(84.6%)	
Partial cystectomy	196(1.0%)	3(1.2%)		8(3.3%)	3(1.2%)	
Radical cystectomy	428(2.2%)	11(4.3%)		6(2.5%)	11(4.3%)	
Pelvic exenteration	443(2.3%)	15(5.9%)		7(2.9%)	15(5.9%)	
No surgery	440(2.3%)	10(3.9%)		12(4.9%)	10(3.9%)	
**Radiotherapy**			0.489			0.387
No/unknown	19172(98.2%)	248(97.6%)		235(96.3%)	248(97.6%)	
Yes	348(1.8%)	6(2.4%)		9(3.7%)	6(2.4%)	
**Chemotherapy**			0.206			0.502
No/unknown	14941(76.5%)	203(79.9%)		189(77.5%)	203(79.9%)	
Yes	4579(23.5%)	51(20.1%)		55(22.5%)	51(20.1%)	
**Tumor size**			0.003*			0.087
≤ 3 cm	5328(27.3%)	47(18.5%)		43(17.6%)	47(18.5%)	
3-6 cm	3918(20.1%)	45(17.7%)		32(13.1%)	45(17.7%)	
> 6cm	687(3.5%)	10(3.9%)		3(1.2%)	10(3.9%)	
Unknown	9587(49.1%)	152(59.8%)		166(68.0%)	152(59.8%)	
**Number of tumors**			0.816			< 0.001*
Single	11285(57.8%)	145(57.1%)		57(23.4%)	145(57.1%)	
Multiple	8235(42.2%)	109(42.9%)		187(76.6%)	109(42.9%)	
**Survival time(month)**			< 0.001*			< 0.001*
Mean	50.69	44.08		54.54	44.08	
Median (25th–75th percentile)	50(30–72)	43(15.75–68.25)		53(26.25–83)	43(15.75–68.25)	

*PUI* prostatic urethra involvement, *TURBT* Transurethral Bladder Tumor Resection, *AIAN* American/Indian/Alaska/Native, *API* Asian/Pacific Islander, *PSM* Propensity score matching

^*^Statistically significant

The External validation cohort included a total of 159 bladder cancer patients. Among them, 152 cases belonged to the non-involved group, while 7 cases were classified under the PUI group. Compared to the non-involved group, the PUI group showed a significantly higher percentage of patients who experienced fatal outcomes (100% vs. 34.9%, *P* = 0.044). Additionally, the survival time in the PUI group was significantly shorter than that of the non-involved patients (5.29 vs. 54.69, *P* < 0.001) (Supplementary Table 2).

### Survival analyses in the matched groups

**Fig. 1:**
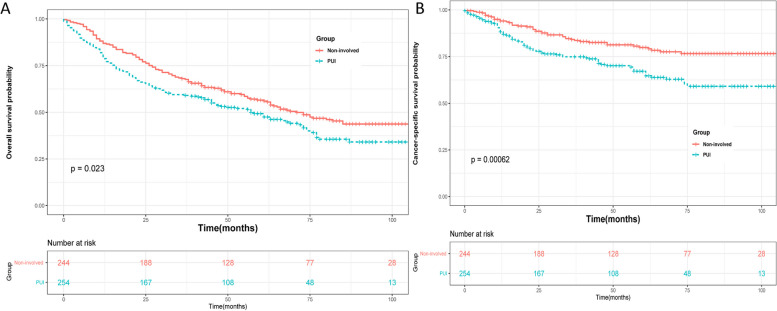
Cancer-specific survival and overall survival of T1 bladder cancer patients in PUI group and Non-involved group after PSM

**Fig. 2:**
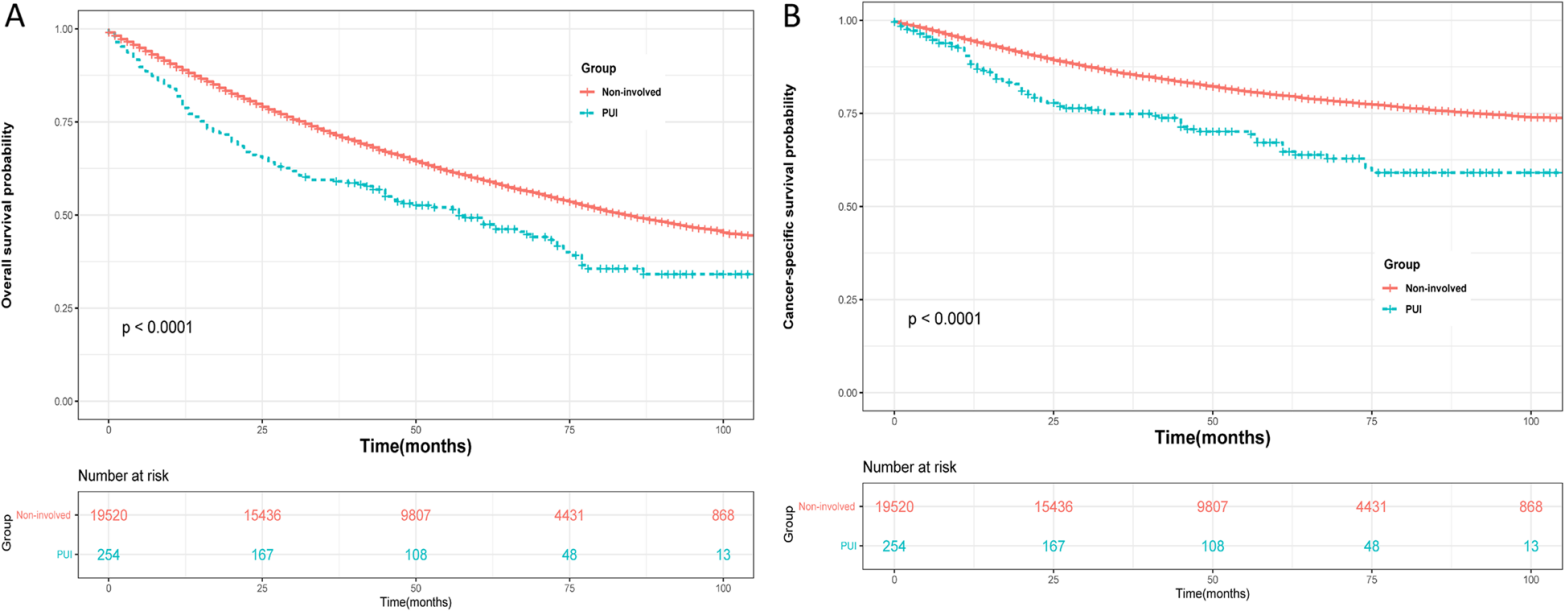
Cancer-specific survival and overall survival of T1 bladder cancer patients in PUI group and Non-involved group before PSM

**Fig. 3:**
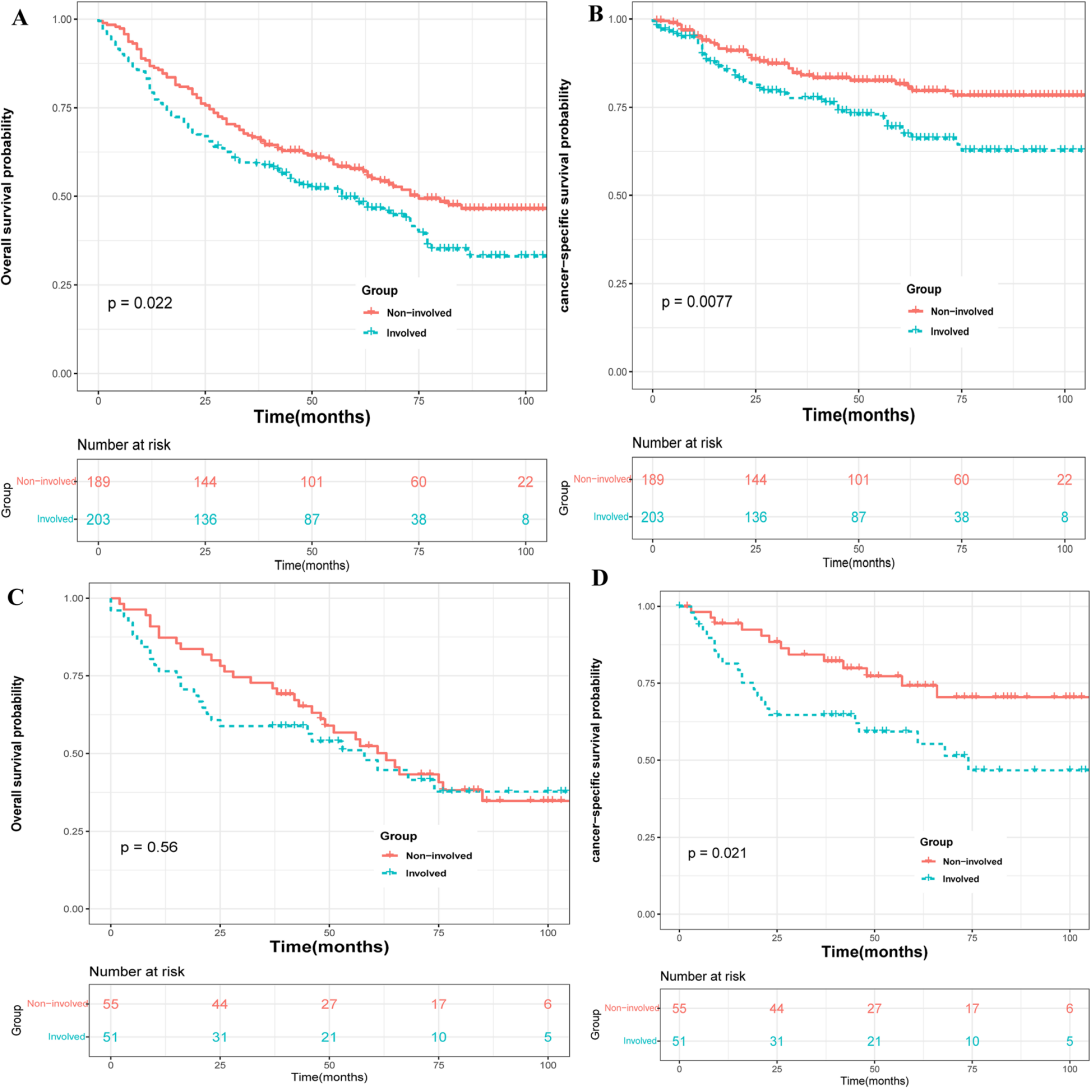
subgroup analyses of T1 bladder cancer patients after PSM based on whether patients received chemotherapy to compare survival outcome between PUI group and Non-involved group: **A** without chemotherapy; overall survival, **B** without chemotherapy; cancer-specific survival, **C** receiving chemotherapy; overall survival, **D** receiving chemotherapy; cancer-specific survival

The median follow-up time was 50 months, and a total of 8412 (43.09%) and 143 (56.3%) deaths from all causes were recorded in the non-involved group and PUI group, respectively. Among these deaths, 3588 (18.38%) and 77 (30.31%) patients specifically died from bladder cancer in the non-involved and PUI groups, respectively. Kaplan–Meier analysis revealed that after propensity score matching (PSM), the PUI group had significantly lower overall survival (OS) and cancer-specific survival (CSS) probabilities compared to the non-involved group (Fig. 1; *P* = 0.023 for OS; *P* < 0.001 for CSS). Similar results were found before PSM (Fig. 2). Subgroup analyses based on whether patients received chemotherapy showed that the PUI group had worse OS and CSS outcomes than the non-involved group in patients without chemotherapy (Fig. 3). Among patients receiving chemotherapy, those with PUI had worse CSS compared to those without. Univariate and multivariate Cox regression analyses were conducted to identify prognostic factors for cancer-specific mortality in patients with T1BC. The results indicated that age, marital status, PUI involvement, tumor size, surgery, and radiation were associated with CSS. Additional details can be found in Supplementary Table 2.

After adjusting for age, race, marital status, grade, tumor size, and number of tumors in model I, PUI was confirmed as an independent risk factor for all-cause mortality (HR: 1.446, 95% CI: 1.210–1.726, *P* < 0.001) and cancer-specific mortality (HR: 1.816, 95% CI: 1.423–2.318, *P* < 0.001) in the multivariate analyses (Table 2). Similar results were obtained in model II, which additionally accounted for surgery, radiotherapy, and chemotherapy (HR: 1.470, 95% CI: 1.231–1.756, *P* < 0.001 for all-cause mortality; HR: 1.817, 95% CI: 1.423–2.320, *P* < 0.001 for cancer-specific mortality). After 1:1 PSM, multivariate Cox regression analyses showed a significant statistical difference in cancer-specific mortality (HR: 1.892, 95% CI: 11.255–2.852, *P* = 0.002 for model I; HR: 2.114, 95% CI: 1.382–3.233, *P* = 0.001 for model II) and all-cause mortality (HR: 1.520, 95% CI: 1.156–2.000, *P* = 0.003 for model I; HR: 1.618, 95% CI: 1.224–2.141, *P* = 0.001 for model II) between the PUI and non-involved groups.

**Table 2:** Univariate and multivariable Cox proportional hazard model for PUI

Outcomes	PUI HR (95% CI) (Non-involved ref.)	*P-*value
**All-cause mortality**		
Non-adjusted	1.495(1.268–1.764)	< 0.001*
Adjusted model I	1.446(1.210–1.726)	< 0.001*
Adjusted model II	1.470(1.231–1.756)	< 0.001*
PSM non-adjusted	1.320(1.037–1.680)	0.024*
PSM adjusted model I	1.520(1.156–2.000)	0.003*
PSM adjusted model II	1.618(1.224–2.141)	0.001*
**Cancer-specific mortality**		
Non-adjusted	1.880(1.500–2.357)	< 0.001*
Adjusted model I	1.816(1.423–2.318)	< 0.001*
Adjusted model II	1.817(1.423–2.320)	< 0.001*
PSM non-adjusted	1.870(1.297–2.696)	0.001*
PSM adjusted model I	1.892(1.255–2.852)	0.002*
PSM adjusted model II	2.114(1.382–3.233)	0.001*

Non-adjusted: Univariate cox regression analysis for PUI

model I adjusted for: Age, race, marital status, grade, tumor size, number of tumors

model II adjusted for: Age, race, marital status, grade, tumor size, number of tumors, surgery, radiotherapy, chemotherapy

*PUI* Prostatic urethra involvement, *PSM* Propensity score matching

^*^Statistically significant

### Building and validating the nomogram for CSS

**Fig. 4:**
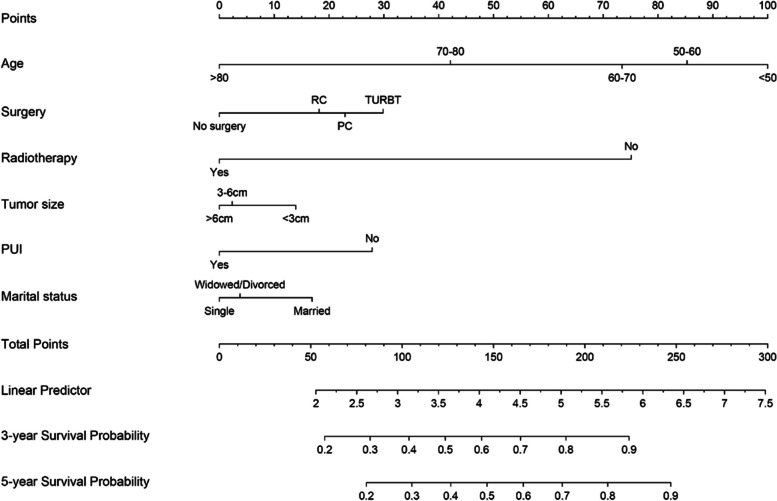
Nomogram predicting 3- and 5-year bladder cancer-specific survival probability for T1 bladder cancer patients. Variables include age, histology, surgery, radiotherapy, tumour size, PUI, and marital status. use: locate patient values at each axis. Draw a vertical line to the ‘‘Point’’ axis to determine how many points are attributed for each variable value. Sum the points for all variables. Locate the sum on the ‘‘Total Points’’ line. Draw a vertical line towards the 3Yrs.Surv. Prob. and 5Yrs.Surv. Prob, Prob. axes to determine respectively the 3-, and 5-year survival probabilities

A predictive model was used to estimate the 3- and 5-year cancer-specific survival (CSS) rates for patients with T1 bladder cancer. The results were visually presented as a nomogram and further validated in a separate group of patients (Fig. 4). The nomogram incorporated six risk factors known to be associated with CSS, namely age, surgery, radiotherapy, tumor size, primary tumor site, and marital status. Age was found to have the highest impact on CSS prognosis.

**Fig. 5:**
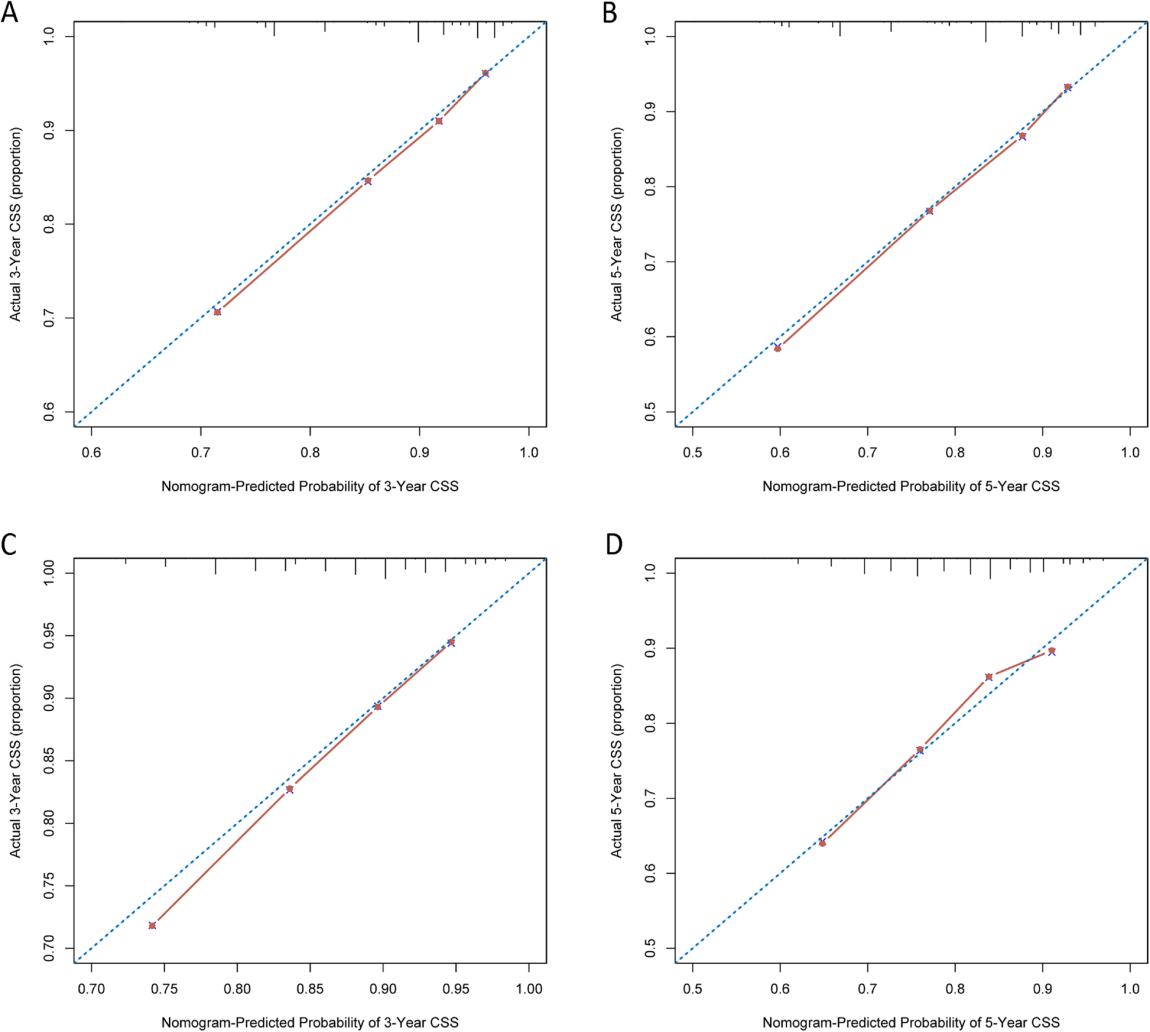
The development cohort **A** Calibration plots of the nomogram for 3-year; **B** Calibration plots of the nomogram for 5-year; The validation group **C** Calibration plots of the nomogram for 3-year; **D** Calibration plots of the nomogram for 5-year

The nomogram demonstrated a relatively good predictive ability for CSS, with a C-index of 0.715 (0.711–0.719) in the development cohort and 0.672 (0.667–0.677) in the validation cohort. The calibration curves (Fig. 5) showed a consistent match between actual observations and predicted outcomes for the probability of 3- and 5-year CSS, indicating good performance and reliability of the model.

## Discussion

Upon extensive analysis of relevant data, it had been observed that T1 bladder cancer patients with PUI exhibit a significantly lower OS and CSS in comparison to patients who do not display PUI (*P* < .05). Additionally, those with PUI were found to have an elevated risk of ACM and CSM, affirming the independent risk factor status of PUI. We also have constructed a predictive model that can provide an accurate estimation of individualized cancer-specific survival probability for T1 bladder cancer patients.

A previous retrospective study has indicated that prostatic urethral involvement may be an independent risk factor for all-cause mortality in patients with non-muscle invasive bladder cancer (NMIBC), A previous retrospective study has indicated that prostatic urethral involvement may be an independent risk factor for all-cause mortality in patients with NMIBC, with inferior recurrence-free, cancer-specific, and overall survival rates observed in patients with prostatic urethral involvement [16]. Similarly, there was an analysis of 146 patients who were confirmed with T1G3 stage concluded that having CIS in the prostatic urethra was concerning the higher risk of recurrence, progression, and CSM [17]. However, the number of patients in the study was insufficient to do more statistical analysis like propensity score matching (PSM). In addition, it might not be suitable to treat Ta, Tis, and T1 bladder tumours as a whole, considering the heterogeneity between them. Similar to our study, we attempted to investigate the prognostic significance of tumors extending to the prostatic urethra and found the worse disease outcome in these patients. Some conjectures based on these results were constructed that whether survival outcome differences existed between PUI and non-involved patients. Unlike previous studies, our study only focused on bladder cancer patients diagnosed with T1 stage because the current guidelines on the treatment of T1 bladder cancer patients were still ambiguous, and convincing shreds of evidence selecting T1 patients to undergo radical cystectomy were lacking [18, 19].

Tumours in the prostatic urethra were difficult to detect in consideration of transurethral biopsy that was not routinely taken in the surgery of TURBT. So diagnostic transurethral biopsy and radical cystectomy removing prostate and bladder were the two main diagnosis methods [9, 16]. In our study, most patients with PUI were confirmed with the method of diagnostic transurethral biopsy due to the utilization of radical cystectomy for T1 bladder cancer patients being relatively rare in our study (10.2%). Notably, according to a single-center retrospective study, roughly 25% of high-grade non-muscle invasive bladder cancer patients were diagnosed with the PUI using the transurethral biopsy when recrudescent bladder tumours were found [9]. However, these patients did not receive the transurethral biopsy, and we failed to know whether the tumor in the prostatic urethra was surgical residue or a recurring tumor. Therefore, relatively a part of patients had a tumor in the prostatic urethra but was neglected. In addition, cystoscopy was the primary approach to detect recrudescence, while the utilization of the transurethral biopsy in the cystoscopy procedure was relatively infrequent [9]. Therefore, PUI might be a common and easily overlooked problem for bladder cancer patients. The first cystoscopy was often performed in the period of three months after TURBT. The transurethral biopsy of the prostatic urethra as a regular operation in the first cystoscopy might be considerable and beneficial for patients.

Furthermore, our study established and internally validated a highly accurate and discriminating nomogram based on risk factors associated with CSS, including demographic, tumour characteristics, and treatment data for T1 bladder cancer patients. The risk factors were filtered by the univariate and multivariate Cox regression analysis. This method finally generated a highly accurate and informative model, which only incorporated influential variables without sacrificing accuracy. Compared to the EORTC and CUETO7 prognostic models, our developed prognostic nomogram is specifically designed to predict the cancer-specific survival (CSS) of T1 bladder cancer patients, whereas the EORTC and CUETO7 models were developed for non-invasive and BCG-treated patients. We could acquire the exact probability of CSS for T1 bladder cancer at a specific time like 3-year and 5-year. Compared to the traditional using a notion of relative risk, the nomogram could quantify risk and give a convincing result. Furthermore, the nomogram model was made to the risk referring to characteristics of personal cancer, which was more closely related to the patient. Moreover, the nomogram's advantage was various assessment methods like the C-index and the calibration curve to evaluate the predictive model’s performance and applicability [20, 21]. Several nomograms, which were used to predict survival outcomes of patients with bladder cancer, had been constructed [22, 23]. However, a prediction model specially designed for T1 bladder cancer patients was imperative regarding varied prognoses and controversy on the treatment for T1 bladder cancer patients [4]. Precise prediction could play a role in individual patient counselling and follow-up schedule.

This study presented several noteworthy strengths. Firstly, we successfully recruited a substantial sample size of 20,370 patients with T1 bladder cancer by utilizing the SEER database, facilitating thorough and comprehensive analysis. Moreover, we employed PSM methodology to address potential selection bias and confounding variables. Additionally, we developed a highly accurate and informative nomogram tailored specifically to assist in treatment decisions and patient counseling for individuals with T1 bladder cancer. However, some limitations should be acknowledged. Firstly, being a retrospective cohort design, our study may have been susceptible to selection bias. Furthermore, even after PSM adjustment, important factors like age, grade, and tumor quantity remained statistically significant. Additionally, vital factors such as lymphatic vessel invasion, detailed pathological records, diagnostic methods, and more information on tumor grade were not available in the SEER database. Furthermore, the number of patients with prior urinary infections (PUI) was relatively low compared to those without (254 vs 19,520). Finally, although our nomograms exhibited impressive accuracy, external and prospective validation is necessary before widespread application.

## Conclusion

The prognosis of T1 bladder cancer involving prostatic urethra was worse for survival outcome than those non-involved. Prostatic urethra involvement was an independent risk factor of ACM and CSM in T1 bladder cancer patients. In addition, we constructed a highly discriminative and precise nomogram that predicted the individualized probability of cancer-specific survival for patients with T1 bladder cancer. This nomogram could work on the decision on treatment, patient counselling, and follow-up schedule for T1 bladder cancer patients.

### Supplementary Information

**Additional file 1: Supplement Table 1.** Basic characteristics of bladder cancer patients with T1 stage in the First Affiliated Hospital of Nanchang University.

**Additional file 2: Supplementary Table 2.** Univariate and multivariate regression analyses for CSM after PSM.

## Data Availability

The data in this article comes from the SEER database This data can be found here: //seer.cancer.gov/data-software/documentation/seerstat/nov2020/.
