# Supplementary material for: Assessing the prognostic impact of prostatic urethra involvement and developing a nomogram for T1 stage bladder cancer
Source: BMC Urol. 2023 Nov 10;23:182. doi: 10.1186/s12894-023-01342-2 (PMC10638768; doi:10.1186/s12894-023-01342-2)
Supplement: Supplementary file 1 — Additional file 1: Supplement Table 1. Basic characteristics of bladder cancer patients with T1 stage in the First Affiliated Hospital of Nanchang University. [file 12894_2023_1342_MOESM1_ESM.docx]

| **Supplement table 1:** **Basic characteristics of bladder cancer patients with T1 stage in the First Affiliated Hospital of Nanchang University** | | | |
| --- | --- | --- | --- |
|  | **Non-involved**  **(N=152)** | **PUI**  **(N=7)** | **P-value** |
| **Age (year)** |  |  |  |
| Mean±SD | 70.6(9.37) | 79(5.22) | 0.02 |
| Median | 70 | 79 | 0.019 |
| **Sex** |  |  | 0.192 |
| Male | 122(80.3%) | 7(100%) |  |
| Female | 30(19.7%) | 0(0%) |  |
| **Grade** |  |  | 0.95 |
| Low | 23(15.1%) | 1(14.3%) |  |
| High | 129(84.9%) | 6(85.7%) |  |
| **Chemotherapy** |  |  | 0.135 |
| no | 147(96.7%) | 6(85.7%) |  |
| yes | 5(3.3%) | 1(14.3%) |  |
| **Status** |  |  | 0.044 |
| Alive | 99(65.1%) | 0(0%) |  |
| Death | 53(34.9%) | 7(100%) |  |
| **Survival month** | 54.69 | 5.29 | <0.001 |
| PUI: prostatic urethra involvement, SD: Standard deviation. | | | |
