# Supplementary material for: Assessing the prognostic impact of prostatic urethra involvement and developing a nomogram for T1 stage bladder cancer
Source: BMC Urol. 2023 Nov 10;23:182. doi: 10.1186/s12894-023-01342-2 (PMC10638768; doi:10.1186/s12894-023-01342-2)
Supplement: Supplementary file 2 — Additional file 2: Supplementary Table 2. Univariate and multivariate regression analyses for CSM after PSM. [file 12894_2023_1342_MOESM2_ESM.docx]

**Supplementary Table 2 Univariate and multivariate regression analyses for CSM after PSM**

|  | **Univariate** |  |  | **Multivariate** |  |  |
| --- | --- | --- | --- | --- | --- | --- |
| **Characteristic** | **HR** | **95% CI** | ***P*** | **HR** | **95% CI** | ***P*** |
| **Age (year)** |  |  |  |  |  |  |
| >60 | Ref. |  | <0.001^※^ | Ref. |  |  |
| <60 | 0.333 | 0.305-0.363 |  | 0.034 | 0.312-0.372 | <0.001^※^ |
| **Race** |  |  |  |  |  |  |
| White | Ref. |  |  | Ref. |  |  |
| Black | 1.148 | 1.056- 1.249 | 0.001^※^ | 1.01 | 0.83- 1.230 | 0.5 |
| AIAN | 0.880 | 0.573- 1.350 | 0.557 | 0.911 | 0.593- 1.399 | 0.670 |
| API | 0.791 | 0.709- 0.883 | <0.001^※^ | 0.812 | 0.727- 1.12 | 0.39 |
| **Marital status** |  |  |  |  |  |  |
| Married | Ref. |  |  | Ref. |  |  |
| Single | 1.157 | 1.080-1.240 | <0.001^※^ | 1.262 | 1.177-1.353 | <0.001^※^ |
| Widowed/Divorced | 1.100 | 1.602-1.762 | <0.001^※^ | 1.595 | 1.517-1.676 | <0.001^※^ |
| **Extension** |  |  |  |  |  |  |
| Non-involved | Ref. |  |  | Ref. |  |  |
| PUI | 1.880 | 1.500-2.357 | <0.001^※^ | 1.817 | 1.423-2.320 | 0.001^※^ |
| **Grade** |  |  |  |  |  |  |
| Low | Ref. |  |  | Ref. |  |  |
| High | 1.387 | 1.300- 1.480 | <0.001^※^ | 1.152 | 0.87- 1.41 | 0.07 |
| **Tumor size** |  |  |  |  |  |  |
| ≤3cm | Ref. |  |  | Ref. |  |  |
| 3-6cm | 1.171 | 1.099-1.247 | <0.001^※^ | 1.152 | 1.082-1.228 | <0.001^※^ |
| >6cm | 1.500 | 1.344-1.674 | <0.001^※^ | 1.464 | 1.311-1.634 | <0.001^※^ |
| **Surgery** |  |  |  |  |  |  |
| TURBT | Ref. |  |  | Ref. |  |  |
| Partial cystectomy | 0.768 | 0.612-0.965 | 0.023^※^ | 0.779 | 0.620-0.978 | 0.031^※^ |
| Radical cystectomy | 0.675 | 0.578-0.788 | <0.001^※^ | 0.664 | 0.569-0.776 | <0.001^※^ |
| No surgery | 1.512 | 1.352-1.709 | <0.001^※^ | 1.299 | 1.152-1.465 | <0.001^※^ |
| **Radiation recode** |  |  |  |  |  |  |
| no/unknown | Ref. |  |  | Ref. |  |  |
| yes | 2.900 | 2.604-3.231 | <0.001^※^ | 1.918 | 1.709-2.152 | <0.001^※^ |
| **Chemotherapy recode** |  |  |  |  |  |  |
| no/unknown | Ref. |  |  | Ref. |  |  |
| yes | 0.855 | 0.813-0.898 | <0.001^※^ | 0.791 | 0.751-1.433 | 0.14 |
| PUI: prostatic urethra involvement, CI, confidence interval; CSM, cancer-specific mortality; HR, hazard ratio; PSM, propensity score matching; Ref., reference, TURBT: Transurethral Bladder Tumor Resection; AIAN, American/Indian/Alaska/Native; API, Asian/Pacific Islander; *Statistically significant. | | | | | | |
|  | | | | | | |
